# Resveratrol Inhibits Protein Translation in Hepatic Cells

**DOI:** 10.1371/journal.pone.0029513

**Published:** 2011-12-29

**Authors:** Eugenia Villa-Cuesta, Joan M. Boylan, Marc Tatar, Philip A. Gruppuso

**Affiliations:** 1 Department of Ecology and Evolutionary Biology, Brown University, Providence, Rhode Island, United States of America; 2 Department of Pediatrics, Brown University and Rhode Island Hospital, Providence, Rhode Island, United States of America; Fundação Oswaldo Cruz, Brazil

## Abstract

Resveratrol is a plant-derived polyphenol that extends lifespan and healthspan in model organism. Despite extensive investigation, the biological processes mediating resveratrol's effects have yet to be elucidated. Because repression of translation shares many of resveratrol's beneficial effects, we hypothesized that resveratrol was a modulator of protein synthesis. We studied the effect of the drug on the H4-II-E rat hepatoma cell line. Initial studies showed that resveratrol inhibited global protein synthesis. Given the role of the mammalian Target of Rapamycin (mTOR) in regulating protein synthesis, we examined the effect of resveratrol on mTOR signaling. Resveratrol inhibited mTOR self-phosphorylation and the phosphorylation of mTOR targets S6K1 and eIF4E-BP1. It attenuated the formation of the translation initiation complex eIF4F and increased the phosphorylation of eIF2α. The latter event, also a mechanism for translation inhibition, was not recapitulated by mTOR inhibitors. The effects on mTOR signaling were independent of effects on AMP-activated kinase or AKT. We conclude that resveratrol is an inhibitor of global protein synthesis, and that this effect is mediated through modulation of mTOR-dependent and independent signaling.

## Introduction

Resveratrol is a plant-derived polyphenol found in grapes, red wine, and other foods. This compound extends the lifespan of lower organisms (yeast, worms, flies and fish) [1]–[3] and protects rodents from a variety of age-related diseases, including cancer, cardiovascular disease, obesity and diabetes [4]–[8]. Resveratrol is considered a mimetic for some of the beneficial effects of caloric restriction (reduction of food intake without malnutrition), which is the only environmental intervention known to extend longevity in a wide range of organisms [9], [10].

A relationship between extended longevity and decreased translation has been observed in a variety of conditions, including caloric restriction [11], [12] and inhibition of the nutrient–sensing kinase termed mTOR (mammalian Target of Rapamycin) [11], [13]–[18]. In fact, recent studies have shown that continuous administration of rapamycin, a specific inhibitor of mTOR Complex 1 (mTORC1), increases lifespan in mice [19] and flies [18]. mTORC1 is one of the two complexes, the other being mTORC2, that account for signaling via mTOR. mTORC1 responds to growth factors, cellular energy and nutrient status by stimulating, among other processes, the initiation of mRNA translation [20]. This involves mTORC1-mediated phosphorylation of the eukaryotic initiation factor 4E-binding protein 1 (eIF4E-BP1) and ribosomal protein S6 kinase 1 (S6K1). Phosphorylation of eIF4E-BP1 leads to its release from the cap-binding factor eIF4E, thereby upregulating cap-dependent translation [21]. Loss of function of eIF4E-BP or S6K1 retards the aging process in flies and mice [12], [17], [18], suggesting that attenuation of signaling through a single mTOR target is sufficient to extend longevity.

mTOR signaling can be activated in response to the serine-threonine kinase AKT. In response to insulin, phosphatidylinositol-3 kinase (PI3K) is activated, leading to the activation of phosphoinositide-dependent kinase-1 (PDK-1), which in turn phosphorylates AKT at Thr 308. Resulting activation of AKT inhibits the formation of the tuberous sclerosis comple/2 (TSC) and de-represses mTORC1 activity [22]. Conversely, low cellular energy levels suppress mTORC1 activity via activation of AMP-activated kinase α (AMPKα). AMPKα activation mediates TSC2 phosphorylation, which results in down regulation of mTORC1 activity [22]. In addition, AMPKα is also able to directly phosphorylate the mTORC1 binding partner Raptor [23]. Interestingly, metformin, an activator of AMPK signaling [22], has been shown to increase longevity and decelerate cancer formation in mice [24], [25].

Among the human disorders that involve dysregulation of mTOR signaling is cancer. Until recently, rapamycin was the only known mTOR inhibitor. However, the acquired resistance of many tumors to rapamycin prompted studies that led to the recent discovery of other mTOR inhibitors [26], including Torin1 and pp242. These inhibitors target the mTOR kinase itself, thereby blocking signal transduction by both mTORC1 and mTORC2.

Until now, the effects of resveratrol and mTOR signaling on longevity and cancer have been considered independent, although mTOR signaling has been shown to be involved in both [11], [13]–[18], [27]–[31]. In the present studies, we examined the effect of resveratrol on the control of protein synthesis and the activation of mTORC1 targets in rat hepatoma cells. Our results show that resveratrol can inhibit global translation in association with signaling effects that are both mTORC1-dependent and independent, thus providing a potential link between control of protein synthesis and the salutary effects of resveratrol on aging and related disease states.

## Results

### Resveratrol inhibits protein translation in rat hepatoma cells

**Figure 1:**
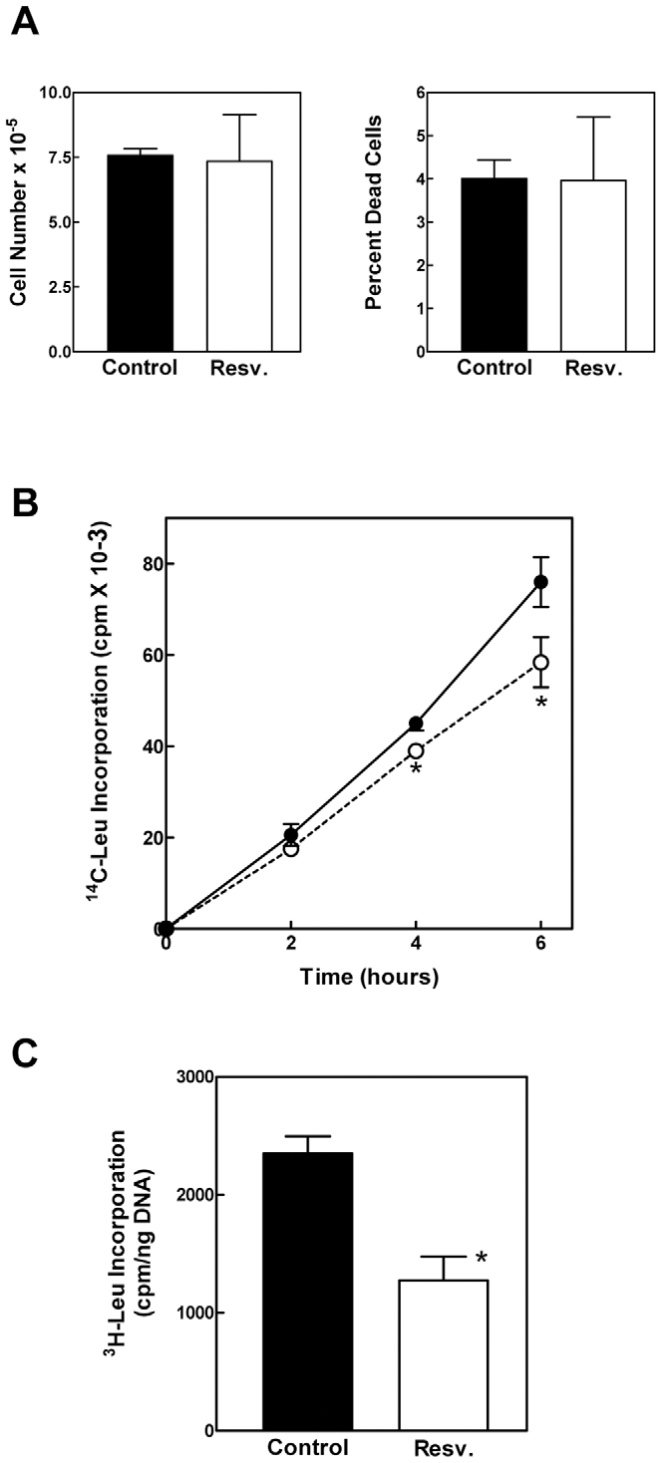
Effect of resveratrol on cell proliferation, viability, and protein synthesis. *Panel A*: H4-II-E hepatoma cells were incubated with DMSO vehicle (control), or resveratrol for 6 hr, at the end of which time total cell number and percentage of dead cells were determined by trypan blue staining and counting. *Panel B*: Cells were incubated with DMSO vehicle (control; filled circles), or resveratrol (unfilled circles) and ^14^C-Leu for 0, 2, 4 or 6 h. At the end of these incubation periods, cell lysates were prepared and analyzed for radiolabeled leucine incorporation into protein. Data, shown as the mean ± SD. *P<0.05 versus control as determined by ANOVA. *Panel C*: ^3^H-Leu incorporation was similarly determined after a 6 h incubation period. Results, normalized to DNA content, are shown as the mean ± SD. *P<0.05 versus control as determined by ANOVA.

High concentrations of resveratrol can induce lysis of human hepatic cells [32]. To avoid such toxicity as a confounding factor, we first determined the maximum resveratrol concentration at which viability of the H4-II-E cells was maintained. Concentrations of resveratrol ranging from 0.01 µM to 500 µM were examined for effects on cell viability. Concentrations up to 5 µM were not associated with changes in cell adherence or morphology. Higher concentrations caused cells to round up and release from the surface of the cell culture plate. Through the use of cell counts and the vital dye trypan blue, we confirmed that 5 µM resveratrol was not associated with reduced cell proliferation or cell viability (Figure 1A).

To determine the effect of resveratrol on protein synthesis, we measured the incorporation of radiolabeled leucine into protein over a time course ranging from 0 to 6 h. ^14^C-Leu incorporation was significantly decreased after cells were treated with 5 µM resveratrol for 4 and 6 h with the value at 2 h not quite reaching statistical significance (Figure 1B). This was interpreted as evidence for a consistent effect of resveratrol on protein synthesis that increased in magnitude with increased duration of exposure. In a repeat experiment in which cells were incubated for 6 h with ^3^H-Leu and resveratrol, protein synthesis was inhibited by approximately 50% when normalized for DNA content (Figure 1C). DNA content was not significantly affected by resveratrol.

One potential caveat in the interpretation of global protein synthesis measurements would be an effect of resveratrol on the transport of leucine into the cell. To test this, cells were exposed to resveratrol for 1 h prior to incubation with ^3^H-Leu for 5, 10 and 15 minutes. At these short time points, ^3^H-Leu incorporation into protein is minimal. In two experiments, cell content of ^3^H-Leu was not significantly affected by resveratrol (Figure S1A), indicating the absence of an effect on leucine transport. In a separate experiment, cells were incubated with ^3^H-Leu for 24 h. The media was removed, the cells were washed, and resveratrol or vehicle was added. ^3^H-Leu remaining in protein was determined after 6 h. Results showed that resveratrol did not affect protein degradation (Figure S1B).

### Resveratrol inhibits mTOR-mediated phosphorylation events

Having determined that resveratrol inhibits protein synthesis in H4-II-E cells, and given the central role of mTOR in translation control, we went on to examine the effect of resveratrol on mTOR signaling. Cells were treated with resveratrol, rapamycin, a specific inhibitor of mTORC1, or pp242, an inhibitor of mTOR kinase activity [26]. Cells were lysed after 1 h. Extracts were analyzed by Western immunoblotting.

**Figure 2:**
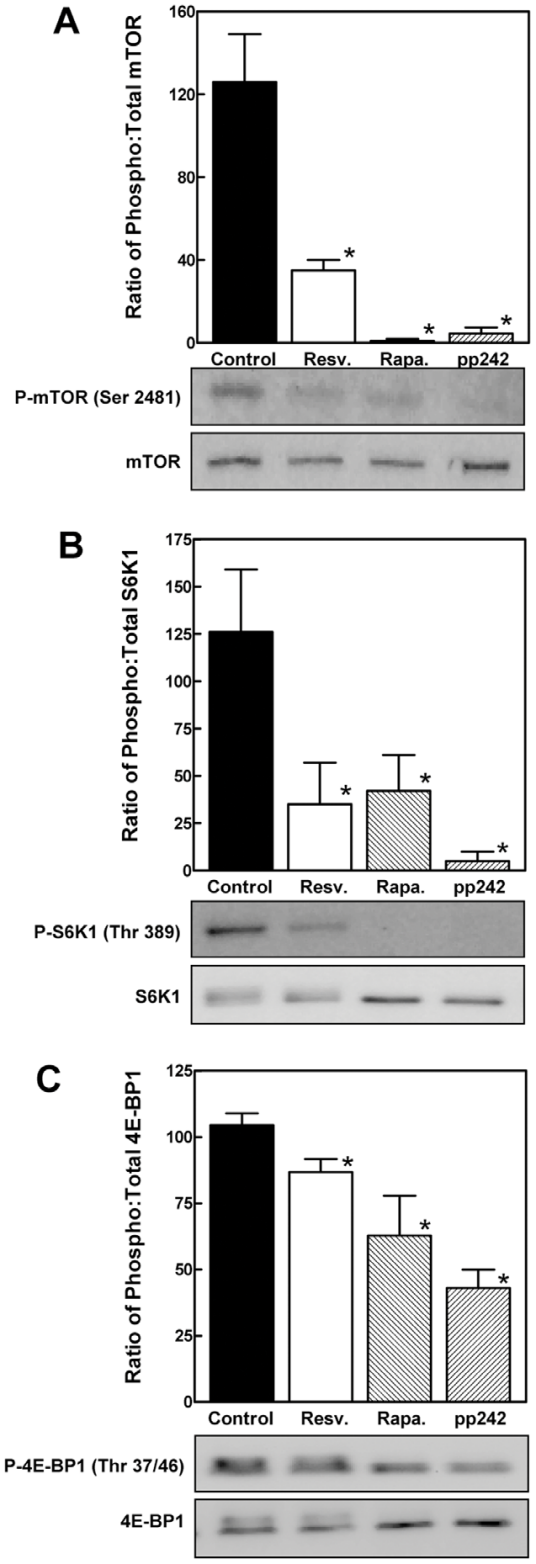
Effect of resveratrol and mTOR inhibitors on mTOR-mediated phosphorylation. H4-II-E hepatoma cells were incubated with DMSO vehicle (control), resveratrol, rapamycin or pp242 for 1 h. Cell lysates were prepared and analyzed by Western immunoblotting. The results of triplicate immunoblots were quantified by densitometry. Data were normalized between blots by analyzing a single control sample on all blots. The results shown in the graphs represent the ratio of phospho∶total mTOR (*Panel A*), S6K1 (*Panel B*) and eIF4E-BP1 (*Panel C*). Representative immunoblots are shown below each graph. Results are shown as the mean ± SD. *P<0.05 versus control as determined by ANOVA.

Resveratrol, rapamycin and pp242 all decreased the phosphorylation of mTOR at a known self-phosphorylation site, Ser 2481 (Figure 2A). Similarly, phosphorylation of S6K1 at an mTOR-dependent site (Thr 389 in the human protein), was inhibited by all three agents (Figure 2B). Phosphorylation of 4E-BP1 at mTOR-dependent sites (Thr 37/46) was reduced significantly, though less markedly, by resveratrol, rapamycin and pp242 treatment (Figure 2C).

### Resveratrol decreases eIF4F complex formation

**Figure 3:**
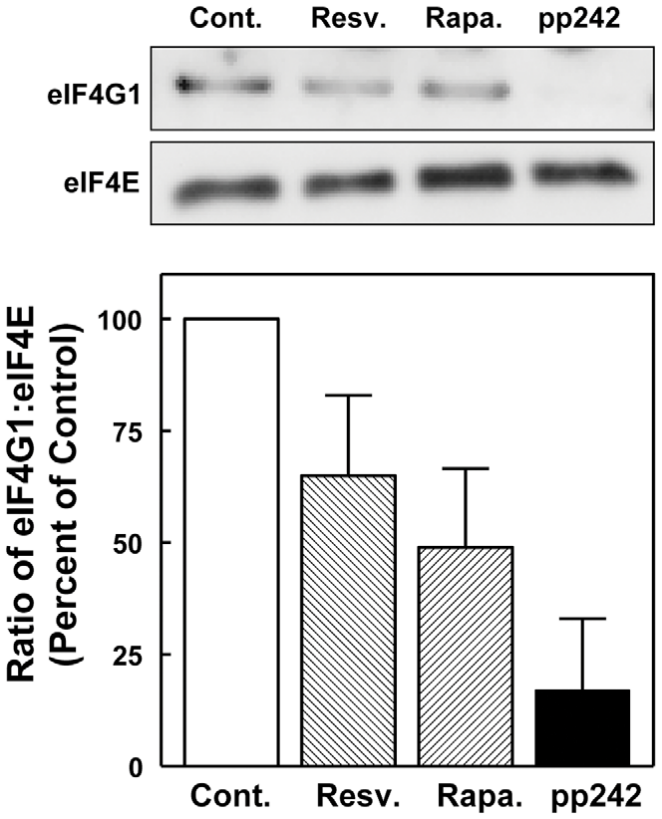
The effect of resveratrol and mTOR inhibitors on the formation of the eIF4F translation initiation translation complex. H4-II-E hepatoma cells were incubated with vehicle (control), resveratrol, rapamycin or pp242 for 1 h. eIF4E-containing complexes were purified from cell lysates using 7mGTP-Sepharose beads. The affinity purified proteins were analyzed by Western immunoblotting using antibodies against eIF4E and eIF4G. Representative immunoblots are shown above the graph. The immunoblots were quantified by densitometry to obtain a ratio of eIF4G to eIF4E for each condition. In order to compare results across experiments, results were expressed as percent of control for each individual experiment and are shown as the mean and standard error of the mean for triplicate determinations.

Initiation of mRNA translation, the primary determinant of translation efficiency, requires the assembling of eukaryotic initiation factors (eIFs) into the multiprotein complex eIF4F. eIF4F includes the cap-recognition factor eIF4E and the scaffold protein eIF4G [33]. To determine if resveratrol affects the formation of the eIF4F complex, cells were incubated with resveratrol, rapamycin or pp242 for 1 h. Cell lysates were subjected to 7mGTP affinity purification of the eIF4F complex. Resveratrol decreased the amount of eIF4G bound to eIF4E (Figure 3). This decrease was similar in magnitude to that observed in cells incubated with rapamycin but less than that resulting from exposure to pp242.

We also examined the phosphorylation of eIF4G1 in the affinity purified eIF4F complexes. Resveratrol exposure was associated with a modest decrease in phosphorylation at Ser 1108, a site that has been shown to be mTORC1-dependent [34] (data not shown).

### Resveratrol–mediated inhibition of mTOR signaling cannot be attributed to effects on AMPK and AKT signaling

Signaling through mTORC1 is modulated in response to upstream signaling involving AMPK and AKT [22]. To assess the effect of resveratrol on these upstream kinases, cell lysates prepared from H4-II-E cells treated with resveratrol were analyzed by Western immunoblotting.

**Figure 4:**
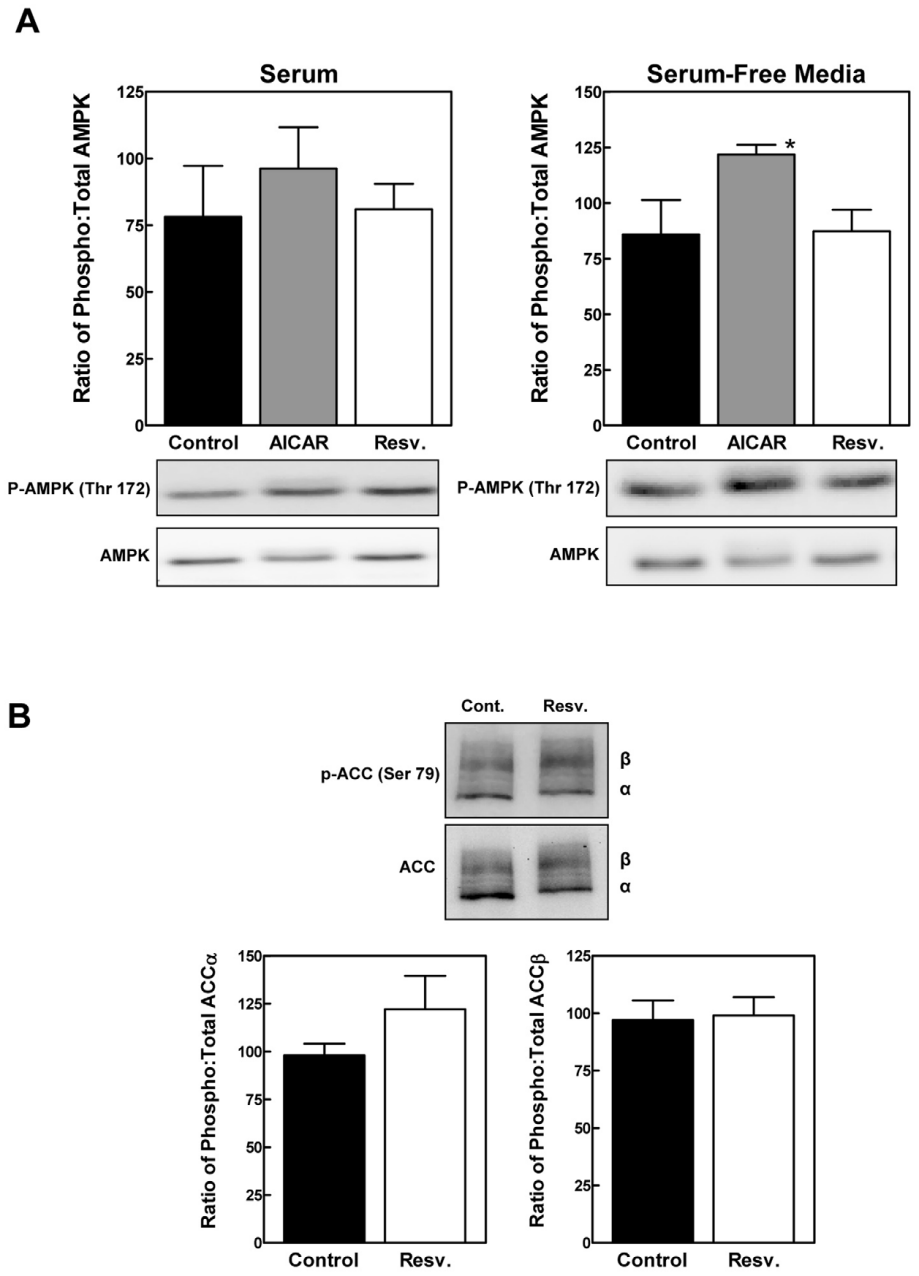
The effect of resveratrol on AMPK activation. *Panel A*: H4-II-E hepatoma cells were incubated with DMSO vehicle (control), resveratrol or AICAR for 1 h in serum containing media (*left*) or serum free media (*right*). Cell lysates were analyzed by immunoblotting for total and phosphorylated AMPKα. *Panel B*: Cells were incubated with DMSO vehicle (control) or resveratrol for 1 h. Samples were analyzed for acetyl CoA carboxylase (ACC) phosphorylation and content. The positions of the α and β ACC isoforms are shown to the right of representative immunoblots. For both panels, the results of triplicate immunoblots were quantified by densitometry. Results are shown as the mean ± SD. *P<0.05 versus control as determined by ANOVA.

We first examined AMPKα (Figure 4A). Using the serum-containing media that were employed for our experiments, phosphorylation of AMPKα was not affected significantly by 0.5 mM of aminoimidazole carboxamide ribonucleotide (AICAR), an AMPKα agonist. Although higher concentrations of AICAR decreased cell viability (data not shown), phosphorylation of AMPKα could be induced by exposure to 0.5 mM of AICAR under serum-free conditions without evidence of cell toxicity, as previously reported [35]. In neither condition was phosphorylation of AMPKα at Thr172 increased in response to resveratrol. Furthermore, resveratrol did not significantly increase phosphorylation of acetyl-CoA carboxylase, a marker of AMPKα signaling (Figure 4B).

**Figure 5:**
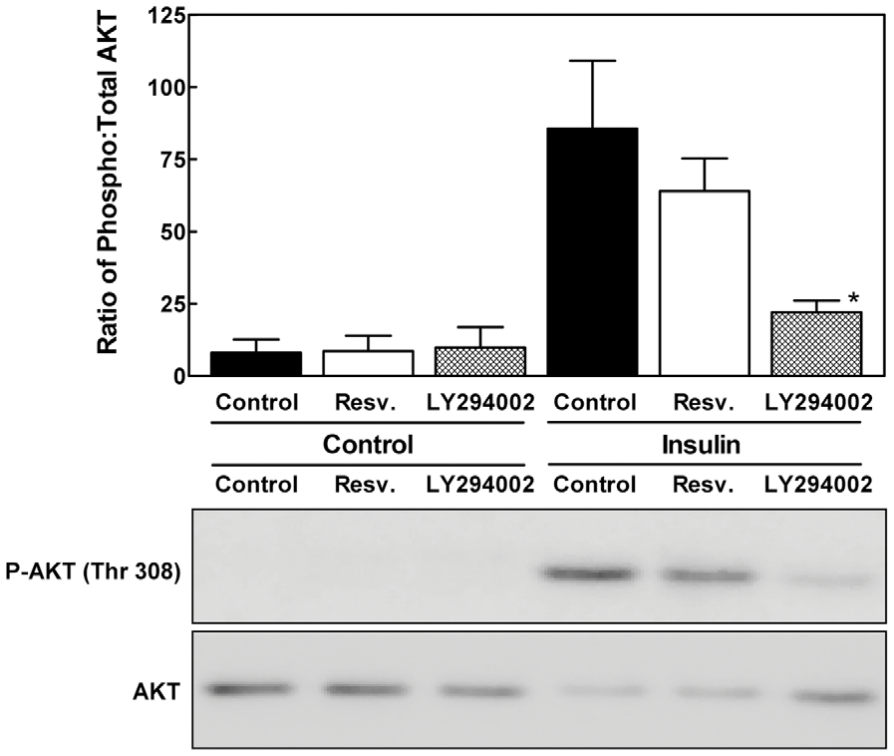
The effect of resveratrol on AKT phosphorylation and the stability of mTORC1. H4-II-E hepatoma cells were incubated with DMSO vehicle (control), resveratrol or LY294002 for 1 h. Where noted, cells were stimulated with insulin 5 min prior to cell lysis. Cell lysates were analyzed by Western immunoblotting for total and phosphorylated AKT at Thr 308. The results of triplicate immunoblots were quantified by densitometry. Data were normalized between blots by analyzing a single control sample on all blots. The results are shown as the mean ± SD. *P<0.05 versus control as determined by ANOVA.

We went on to study signaling involving AKT (Figure 5). Under basal conditions, AKT phosphorylation at the PDK1-dependent Thr308 site was minimal. This was particularly apparent when contrasted with results obtained with cells exposed to 100 nM of insulin for 5 min. The low level of AKT phosphorylation under basal conditions precluded the detection of an inhibitory effect of resveratrol. To determine if resveratrol inhibits the phosphorylation of AKT at Thr308 upon insulin stimulation, we incubated cells with resveratrol or the PI3K inhibitor LY294002 as a control [36]. Insulin-induced phosphorylation of AKT at Thr308 was not reduced significantly by resveratrol. As expected, LY294002 blocked insulin-induced AKT Thr308 phosphorylation. We interpreted these results as indicating that under the conditions employed to examine the effect of resveratrol on basal (non-stimulated) protein synthesis and mTORC1 signaling, AKT was not active. We concluded that inhibition of AKT was not a viable explanation for the aforementioned resveratrol effects.

### Resveratrol does not disrupt mTORC1

**Figure 6:**
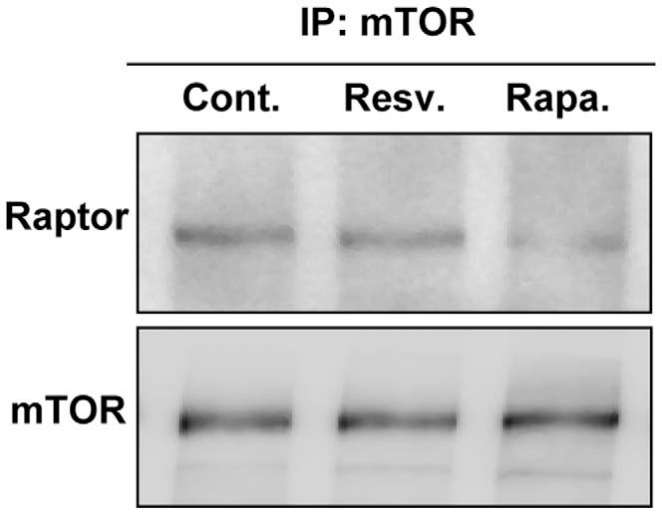
The effect of resveratrol and rapamycin on integrity of mTORC1. Cell lysates were analyzed by immunoprecipitation of mTOR followed by immunoblotting for raptor and mTOR. A second replicate experiment yielded similar results (Figure S3).

Major components of mTORC1 include mTOR itself, raptor and mLST8 [22]. Inhibition of mTORC1 signaling by rapamycin is associated with dissociation of raptor from mTOR [37], [38]. Given that the upstream kinases AKT and AMPKα did not appear to be involved in resveratrol's inhibition of mTORC1, we compared its effect on mTORC1 to that of rapamycin. mTOR was immunoprecipitated from cells exposed to resveratrol and rapamycin. Raptor content in the immunoprecipitated complex was determined by immunoblotting (Figure 6 and Figure S3). Consistent with previous findings [37], [38], rapamycin decreased the amount of the raptor:mTOR complex. This effect was not seen with resveratrol.

Although the interpretation of immunoprecipitation kinase assays for mTOR is complicated owing to the multi-protein complexes in which mTOR resides, we considered the direct inhibition of mTOR kinase activity by resveratrol to be an important mechanism to explore. Using recombinant 4E-BP1 as a phosphate acceptor, we measure kinase activity in mTOR immunoprecipitates from cells cultured under control conditions or exposed to resveratrol. Results (not shown) indicated no effect of resveratrol on mTOR kinase activity.

### Effect of resveratrol on eIF2α phosphorylation

**Figure 7:**
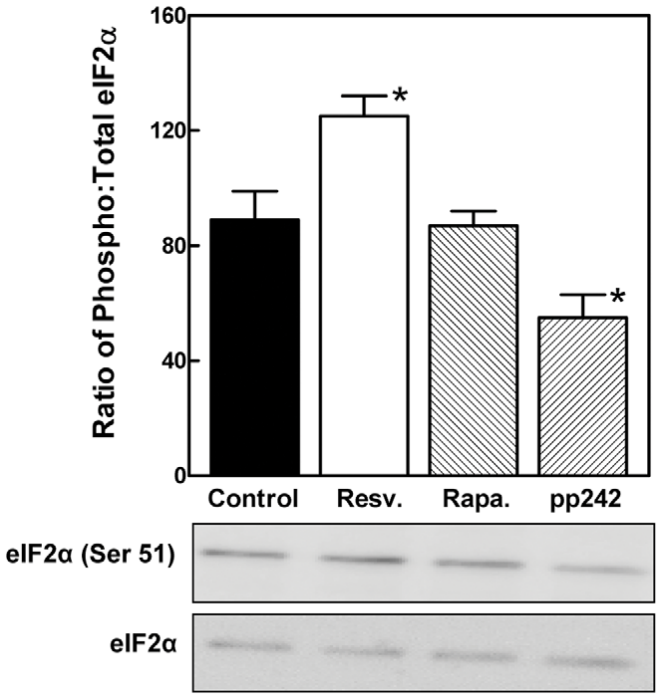
The effect of resveratrol and mTOR inhibitors on eIF2α. H4-II-E hepatoma cells were incubated with DMSO vehicle (control), resveratrol, rapamycin or pp242 for 1 h. Cell lysates were analyzed by Western immunoblotting to determine the ratio of phospho∶total eIF2α. A representative immunoblot is shown below the graph. The results are shown as the mean ± SD. *P<0.05 versus control as determined by ANOVA.

Phosphorylation of eIF2α inhibits the ability of the eIF2 complex to deliver the charged methionyl initiator tRNA to the initiation codon, thereby downregulating translation initiation [39]. eIF2α phosphorylation is mediated by multiple kinases, including GCN2. Those enzymes are not targets of mTOR. Resveratrol increased eIF2α phosphorylation at Ser 51 (Figure 7). In contrast, rapamycin had no effect while pp242 exposure was associated with a decrease in phosphorylation.

## Discussion

While limited information is available regarding the tissue uptake of resveratrol, it is known that the liver plays a key role in the metabolism of dietary polyphenols, including resveratrol. Despite the high absorption of resveratrol administered orally to humans, resveratrol bioavailability is low because it is rapidly metabolized in liver via glucuronate and sulfate conjugation [40]. In fact, unmetabolized resveratrol has a relatively short half life [41]. It has been suggested, therefore, that *in vitro* investigations of resveratrol biological effects should focus on resveratrol conjugates [42]. Due to the abundance of resveratrol conjugates in rat hepatocytes after incubation with resveratrol [43], we consider hepatic cells to be an advantageous model system for the investigation of resveratrol effect. Additionally, hepatic cells take up resveratrol by both passive diffusion and active transport [44]. This may account for the fact that our experiments used resveratrol at ten to twenty times lower concentrations than generally used in other types of cells, such as human embryonic kidney cells [45], cardiac myocytes [46], HeLa cells [47] and mouse embryonic fibroblasts [47].

Hepatoma cells are also a relevant model because resveratrol has been proposed as a chemopreventative agent for human hepatocellular carcinoma [48], [49] a disease in which the mTOR pathway is often constitutively activated [50]. However, the mechanisms by which resveratrol affects cancer development and/or progression remain uncertain [8], [51]–[53].

Inhibition of translation is known to affect numerous physiological functions, including the stress response and oncogenic signaling [54], [55] Interestingly, dietary restriction has been shown to have a dramatic effect on tumor suppression in almost every tumor tested [56] with the exception of tumors with activated PI3K [57]. In addition, dietary restriction [9], [10] and mTOR inhibition, related conditions owing to the biological function of mTOR, increase longevity in a number of model systems. These include yeast [15], worms [11], [13], [16] , flies [14], [18], [58] and mammals [19]. Because resveratrol can extend lifespan in animals and repression of mTOR is associated with retarded aging, we hypothesized that resveratrol could modulate protein synthesis, perhaps through effects on mTOR signaling or other well-characterized mechanisms involved in the control of translation. Our data support this hypothesis.

Our initial studies demonstrated that resveratrol induces a modest inhibition of global translation in H4-II-E hepatic cells. The magnitude of this effect is similar to that observed by us using rapamycin [59]. Resveratrol-induced reduction of global protein synthesis in the H4-II-E cells was independent of any change in cell number or cell viability. Our results identified multiple signaling events that could account for this effect, including inhibition of mTOR activity (as indicated by the mTOR self-phosphorylation site), formation of the eIF4F cap-binding complex, phosphorylation of eIF4G1, and phosphorylation of eIF2α. The last of these effects was not recapitulated by rapamycin, a finding that distinguishes the signaling effects of resveratrol and from those of mTORC1-specific of general mTOR inhibition. It should be noted that our findings do not rule out other effects of resveratrol on translation initiation. In fact, eIF4A has been recently suggested as a direct target of resveratrol binding *in vitro* [60].

The regulation of mTOR signaling is highly complex. It involves large, multi-protein complexes and input from an array of upstream signals [22]. Studies to date have led to the characterization of two mTOR-containing complexes, mTORC1 and mTORC2. The former is known to account for mTOR-mediated translation control and rapamycin sensitivity. Unlike rapamycin, resveratrol did not affect the binding between raptor, a component of mTORC1, and mTOR. On the other hand, we found decreased mTOR self-phosphorylation at Ser 2481. This suggested that resveratrol may directly inhibit mTOR kinase activity, a possibility that was not supported by results of immunoprecipitation kinase assays. However, resveratrol may interact with other components of the mTORC1 complex or modulate the activity of other mTOR effectors. Among these are the upstream kinases AMPK and AKT [61]. The former is especially interesting since AMPK-deficient mice have been described to be resistant to the metabolic effects of resveratrol [62]. However, our studies implicate neither AMPK nor AKT signaling in resveratrol-induced inhibition of mTORC1 signaling.

In the case of AMPK, the basis for our results may lie in the high sensitivity of H4-II-E cells to resveratrol. We performed our studies at 5 µM, a concentration of resveratrol that did not affect cell proliferation or viability. Concentrations of resveratrol reported to activate AMPK are more than twice those used in our experiments [61], [63]–[65]. In addition, H4-II-E cells seem to be particularly resistant to AMPK activation under basal conditions, since nutrient stress (elimination of serum from culture media) was required for AICAR to activate AMPK. Similarly, the basal level of AKT phosphorylation at Thr308, the PDK1 site, was minimal in H4-II-E cells. This was most apparent when compared to cells exposed to insulin, indicating that inhibition of AKT activity is unlikely to account for any of the effects of resveratrol that we observed. An inhibitory effect of resveratrol on phosphorylation of AKT at Ser 473 has been previously observed [66]. Our results indicate that this could be due to an effect of resveratrol on mTOR signaling. However, the inhibitory effect of resveratrol on translation that we observed was seen under basal, not insulin-stimulated, conditions.

A commonly cited mechanism of resveratrol action is the activation of the NAD^+^-dependent protein deacetylase SIRT1 [1], [2]. Given our results on AMPK and AKT signaling, the role of SIRT1 may be of particular importance. The molecular mechanism of resveratrol action through activation of SIRT1 remains to be elucidated [1], [67], [68]. In fact, recent studies suggest that resveratrol-mediated effects *in vivo* may be independent of a direct activation of SIRT1 [69], [70]. More recently, SIRT1 was implicated as a regulator of mTOR signaling [47]. In these studies, SIRT1-deficient Hela cells and mouse embryonic cells were shown to have elevated phosphorylation of S6K1 and 4EB-P1. However, these investigators found that resveratrol could decrease the phosphorylation of the S6K1 target ribosomal protein S6 in cells depleted of SIRT1, although it did so with less efficiency under these conditions [47]. In other studies implicating S6K1 in the mechanism of resveratrol action [45], the effect was again found to be independent of SIRT1. We have performed preliminary studies showing that the inhibition of S6K1 phosphorylation by resveratrol in hepatic cells was not affected by the SIRT1 inhibitor EX527 (Figure S2A). Likewise, EX527 did not block the effect of resveratrol on the phosphorylation of 4EBP1 and mTOR (data not shown). EX527 has been used by numerous investigators as a selective inhibitor of SIRT1 deacetylase at concentrations similar to those that we employed [66], [71]–[73]. However, we were unable to validate an effect of EX527 on SIRT1 in H4-II-E cells. In an attempt to do so, we studied the expression and acetylation of several SIRT1 targets, PGC1α, FoxO1 and p53. Only p53 was detectable by Western blotting under basal conditions (Figure S2B). We detected no acetylation of this or any other SIRT1 target in the presence of EX527 despite employing a variety of conditions. Therefore, we are unable to draw any definitive conclusions regarding the efficacy of EX527 or the role of SIRT1 in the effects we observed. During the preparation of this manuscript, an inhibitory effect of resveratrol on insulin and leucine stimulated mTOR signaling was described in mouse fibroblasts [74]. This effect was found to be independent of Sirt1 activation, instead involving changes in the interaction of mTOR and its inhibitor DEPTOR.

The nutrient-sensing mTOR pathway has emerged as a link between nutrient availability, translation control, longevity and oncogenesis [11], [13]–[18], [27]–[29], [58]. Our findings identify resveratrol as a modulator of protein synthesis, translation initiation and elongation factors, and mTOR signaling. Any or all of these effects could contribute to the anti-aging and anti-carcinogenic properties of resveratrol. Furthermore, we have found that resveratrol decreases mTOR-dependent phosphorylation, although resveratrol and mTOR inhibitors show different effects on the phosphorylation of eIF2α. This increase in eIF2α phosphorylation induced by resveratrol is of particular interest given the ability of amino acid restriction to induce this same effect [75]. Considered in light of the role of amino acids in modulating mTOR signaling [33], we propose that resveratrol may regulate translation through mechanisms similar to those that sense reduced amino acid availability. The use of an immortalized cell line as a model system has limitations with regard to physiologic significance, so *in vivo* studies will be needed to more definitively address the role of resveratrol on protein translation in the liver and in other tissues and organs.

## Materials and Methods

### Materials

Antibodies to mTOR, phospho-mTOR, S6K1, phospho-S6K1, phospho-eIF4E-BP1, eIF4E, AMPKα, phospho-AMPKα, acetyl CoA carboxylase, phospho-acetyl CoA carboxylase, raptor, phospho-eIF2α, acetylated lysine, acetylated-p53, AKT, and phospho-AKT were purchased from Cell Signaling Technology (Danvers, MA, USA.). Antibodies to eIF4E-BP1, eIF4G1, p53, FoxO1, PGC1α, and eIF2α were purchased form Santa Cruz Biotechnology (Santa Cruz, CA, USA.). Protein A-Sepharose CL-4B and 7-methyl-GTP (7mGTP)-Sepharose 4B were purchased form GE Healthcare (Piscataway, NJ, USA.). Resveratrol was a gift from D. Sinclair (Harvard Medical School, Boston, MA, USA.). Rapamycin was purchased from LC Laboratories (Woburn, MA, USA.). pp242 was purchased from Chemdea (Ridgewood, NJ, USA.). EX527 was purchased form TOCRIS Bioscience (Ellisville, MO, USA). ^3^H-Leu (specific activity: 100 to 150 Ci/mmol ) and ^14^C-Leu (specific activity >300 mCi/mmol) were purchased from Dupont/New England Nuclear (Boston, MA, USA). The Quant-iT PicoGreen® dsDNA Assay Kit was purchased from Invitrogen Corporation (Carlsbad, CA, USA.). Protein was quantified using the BCA Protein Assay Kit from Thermo Scientific (Rockford, IL, USA.)

### Biochemical analyses

H4-II-E cells [76] were cultured in Minimal Essential Medium Gibco # 4100-034 (Carlsbad, CA, USA) with 5% Fetal Bovine Serum. The composition of the media, including amino acid content, can be found at http://www.invitrogen.com/site/us/en/home/support/Product-Technical-Resources/media_formulation.99.html. In particular, leucine was present at a concentration of 0.397 mM for all experiments. Resveratrol, rapamycin, pp242 and EX527 were dissolved in 100% DMSO. Control conditions used equivalent DMSO concentrations.

Protein synthesis was measured as the incorporation of ^3^H-Leu or ^14^C-Leu into protein [59]. Cells were plated in six well plates (2×10^5^ per well) and allowed to attach overnight. Resveratrol and radiolabeled leucine (1 µCi/ml) were added and allowed to incubate for designated time periods prior to cell lysis. Where noted, results were normalized to DNA content, which was measured using the fluorescent Quant-iT PicoGreen® dsDNA Assay Kit with λDNA (0–500 ng/ml) as standard.

For leucine uptake experiments, cells were incubated with resveratrol (5 µM) for 1 h prior to addition of ^3^H-Leu (1 µCi/ml). Cells were lysed after 5, 10 or 15 min. Correction for non specific uptake was made by determining the amount of radiolabeled leucine retained in the extracellular space or cell surface after incubation of ^3^H-Leu for 10 seconds. Protein degradation was also assessed, using the method of [77], [78]. To do so, ^3^H-Leu was added to the cells for 24 h. Cells were washed with fresh media and incubated with resveratrol. After 6 h of incubation, cells were washed twice with cold PBS and precipitated with 10% trichloracetic acid (TCA). The percentage of protein degraded over 6 h treatment was calculated as 100D/(D+E+F) where D is the total cpm in the TCA precipitated fraction, E is the total cpm in the TCA non precipitated fraction, and F is the total cpm in the resveratrol media and PBS washes.

Western immunoblotting was carried out as previously described [59], [79]. For 7mGTP cap-binding studies, Western immunoblotting and mTOR immunoprecipitation, cells (2×10^6^ per well) were allowed to attach overnight to 10 cm plates. Resveratrol (5 µM), rapamycin (100 nM) [56], pp242 (100 nM) [80] and EX527 (1 µM or 5 µM) were added to cells for 1 h. In experiments where cells were serum deprived, cells were transferred to serum-free media and exposed to resveratrol (5 µM) or AICAR (0.5 mM) for 1 h. In experiments where cells were stimulated with insulin, cells were incubated with resveratrol (5 µM) or LY294002 (10 µM) for 1 h and exposed to insulin (100 nM) for 5 min prior cell lyses.

For 7mGTP cap-binding analysis, cell lysates were prepared and analyzed as previously described [80]. For mTOR immunoprecipitation, cells were lysed in 0.3% Chaps buffer [38] and sonicated 2 times for 10 seconds. After pre-clearing with protein A-Sepharose CL-4B, 400 µg of cell lysate protein was incubated overnight with mTOR antibody. A 1∶1 suspension of protein A-Sepharose CL-4B (50 µl) in 0.3% Chaps buffer was added to the sample and incubated for 3 h at 4°C. Immunoprecipitates were washed with 0.3% Chaps buffer four times, and samples were resolved and analyzed by immunoblotting.

### Statistical analyses

Except where noted, data are shown as the mean and standard deviation. Two-way comparisons were performed using the unpaired t-test. Analysis of variance with a Tukey post hoc test was used in cases of multiple comparisons. Differences were considered significant at p<0.05. Experiments were independently repeated at least twice with three biological replicates each time.

## Supporting Information

Figure S1 **The effect of resveratrol on leucine uptake and protein degradation.** *Panel A:* Leucine uptake. H4-II-E hepatoma cells were incubated with resveratrol for 1 h prior to addition of ^3^H-Leu. Cells were lysed after 5, 10 or 15 min. Correction for non specific uptake was made by determining the amount of radiolabeled leucine retained in the extracellular space or cell surface after incubation of ^3^H-Leu for 10 seconds. P = 0.119 at 15 minutes. *Panel B:* Protein degradation was assessed by measuring the uptake and subsequent release of ^3^H-Leu. ^3^H-Leu was added to the cells for 24 h. Cells were washed with fresh media and incubated with resveratrol or vehicle control. After 6 h of incubation, cells were washed twice with cold PBS and precipitated with 10% trichloracetic acid (TCA). The percentage of protein degraded over 6 h treatment was calculated as 100D/(D+E+F) where D is the total cpm in the TCA precipitated fraction, E is the total cpm in the TCA non precipitated fraction, and F is the total cpm in the resveratrol media and PBS washes. The results are shown as the mean ± SD. *P<0.05 versus control as determined by ANOVA.
(TIF)

Figure S2 **The effect of EX527 on the effect of resveratrol.** *Panel A*: H4-II-E hepatoma cells were incubated for 1 h with DMSO vehicle or resveratrol (5 µM) in the presence of 0, 1 or 5 µM EX527. Cell lysates were analyzed by immunoblotting for total and phosphorylated S6K1. The Western immunoblots were quantified by densitometry to determine the ratio of phospho∶total S6K1. A representative immunoblot is shown below the graph. The results are shown as the mean ± SD. *P<0.05 versus control as determined by ANOVA. *Panel B*: The cell lysates prepared for the experiment shown in *Panel A* were analyzed by immunoprecipitation of p53 followed by Western immunoblotting for acetylated and total p53.
(TIF)

Figure S3 **Independent repetition of the effect of resveratrol and rapamycin on integrity of mTORC1.** Cell lysates were analyzed by immunoprecipitation of mTOR followed by immunoblotting for raptor and mTOR.
(TIF)
