# Supplementary figures and images for: Resveratrol Inhibits Protein Translation in Hepatic Cells
Source: PLoS One. 2011 Dec 29;6(12):e29513. doi: 10.1371/journal.pone.0029513 (PMC3248458; doi:10.1371/journal.pone.0029513)

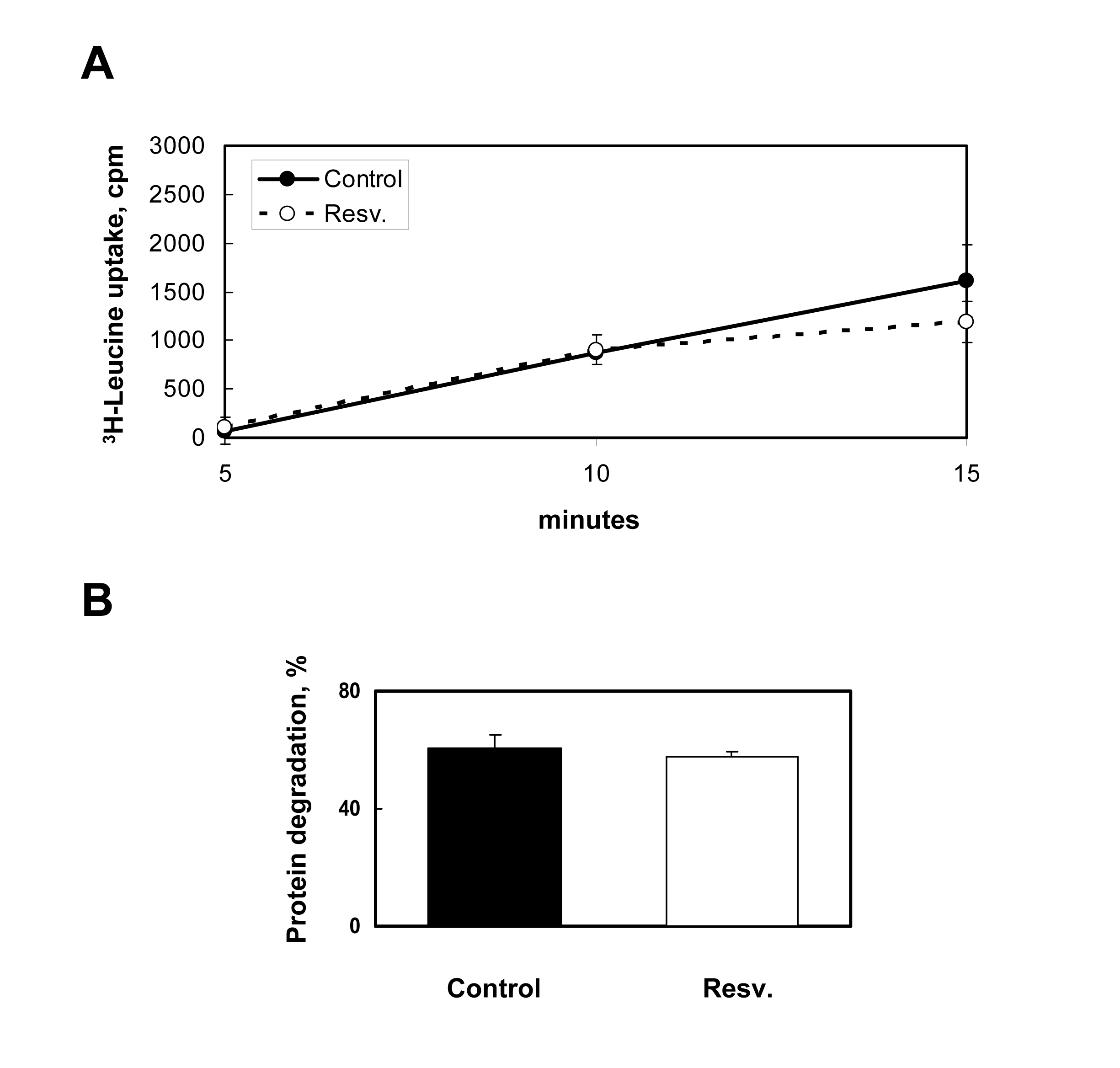

Supplement: Figure S1 — The effect of resveratrol on leucine uptake and protein degradation. Panel A: Leucine uptake. H4-II-E hepatoma cells were incubated with resveratrol for 1 h prior to addition of 3H-Leu. Cells were lysed after 5, 10 or 15 min. Correction for non specific uptake was made by determining the amount of radiolabeled leucine retained in the extracellular space or cell surface after incubation of 3H-Leu for 10 seconds. P = 0.119 at 15 minutes. Panel B: Protein degradation was assessed by measuring the uptake and subsequent release of 3H-Leu. 3H-Leu was added to the cells for 24 h. Cells were washed with fresh media and incubated with resveratrol or vehicle control. After 6 h of incubation, cells were washed twice with cold PBS and precipitated with 10% trichloracetic acid (TCA). The percentage of protein degraded over 6 h treatment was calculated as 100D/(D+E+F) where D is the total cpm in the TCA precipitated fraction, E is the total cpm in the TCA non precipitated fraction, and F is the total cpm in the resveratrol media and PBS washes. The results are shown as the mean ± SD. *P<0.05 versus control as determined by ANOVA. (TIF) [file pone.0029513.s001.tif]

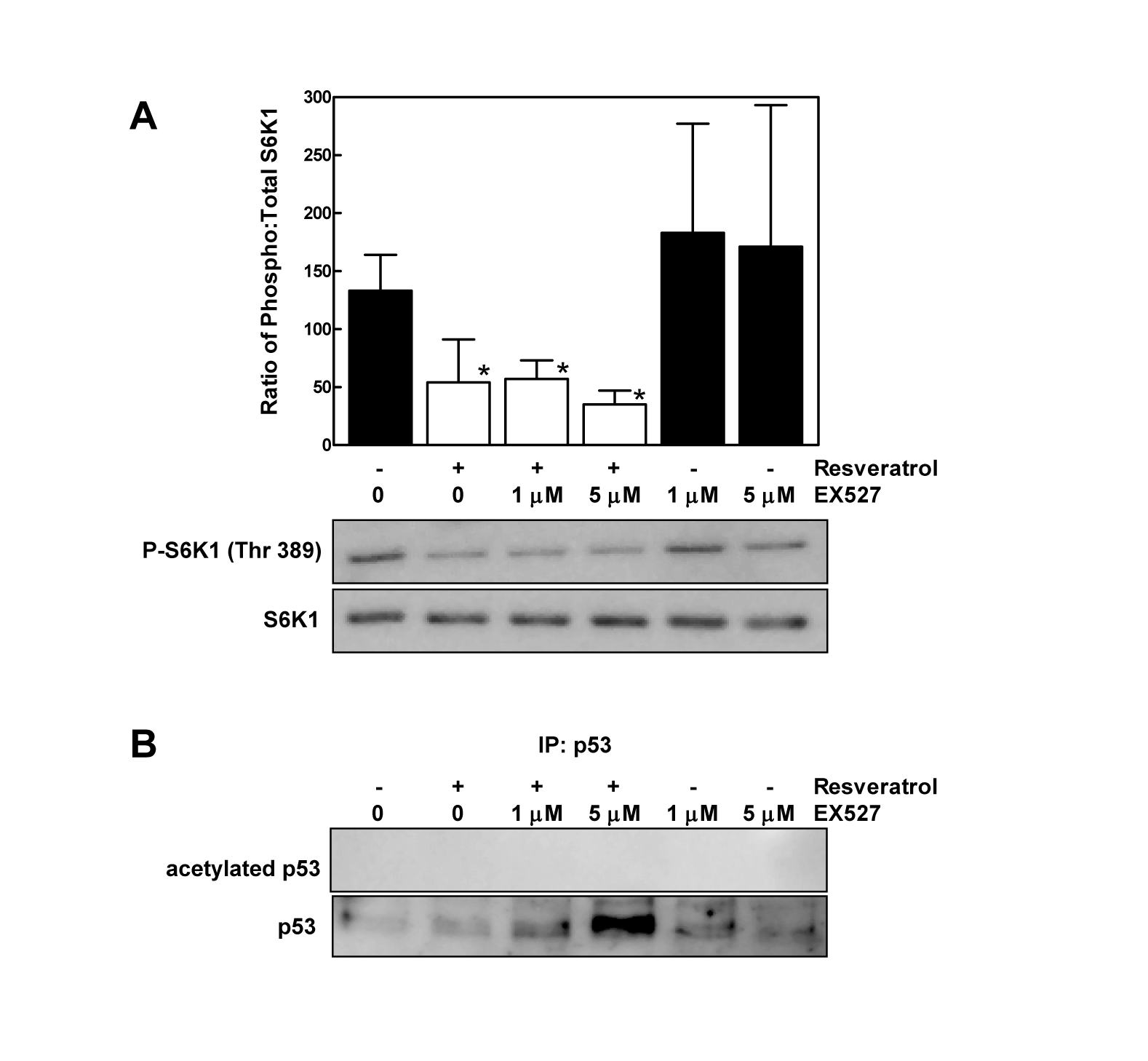

Supplement: Figure S2 — The effect of EX527 on the effect of resveratrol. Panel A: H4-II-E hepatoma cells were incubated for 1 h with DMSO vehicle or resveratrol (5 µM) in the presence of 0, 1 or 5 µM EX527. Cell lysates were analyzed by immunoblotting for total and phosphorylated S6K1. The Western immunoblots were quantified by densitometry to determine the ratio of phospho∶total S6K1. A representative immunoblot is shown below the graph. The results are shown as the mean ± SD. *P<0.05 versus control as determined by ANOVA. Panel B: The cell lysates prepared for the experiment shown in Panel A were analyzed by immunoprecipitation of p53 followed by Western immunoblotting for acetylated and total p53. (TIF) [file pone.0029513.s002.tif]

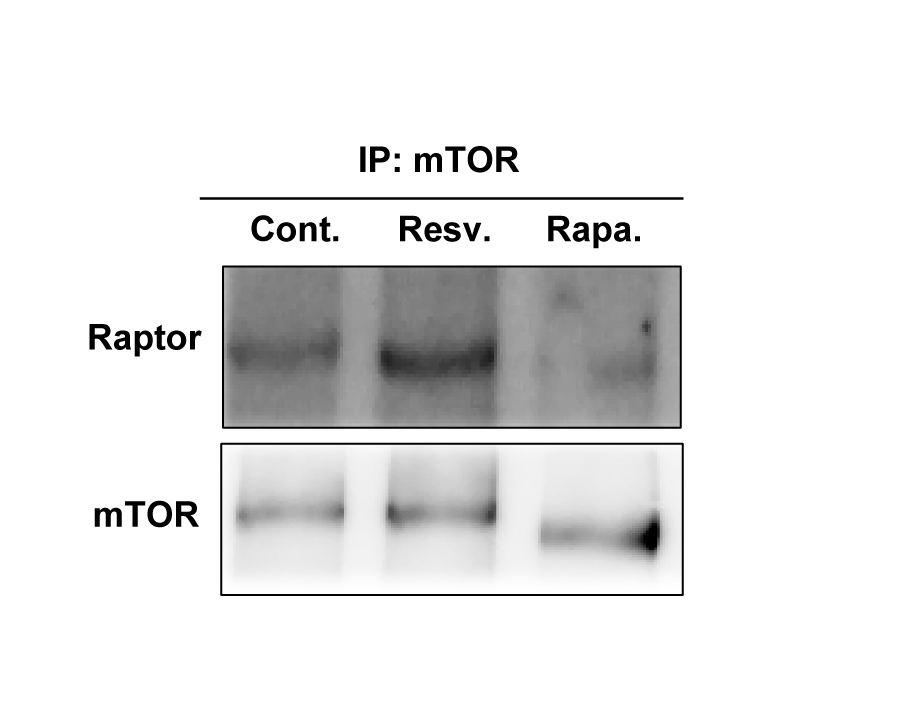

Supplement: Figure S3 — Independent repetition of the effect of resveratrol and rapamycin on integrity of mTORC1. Cell lysates were analyzed by immunoprecipitation of mTOR followed by immunoblotting for raptor and mTOR. (TIF) [file pone.0029513.s003.tif]
